# Fractional Stochastic Differential Equation Approach for Spreading of Diseases

**DOI:** 10.3390/e24050719

**Published:** 2022-05-17

**Authors:** Leonardo dos Santos Lima

**Affiliations:** Federal Center for Technological Education of Minas Gerais, Belo Horizonte 30510-000, MG, Brazil; lslima@cefetmg.br

**Keywords:** fractional Brownian motion, spreading

## Abstract

The nonlinear fractional stochastic differential equation approach with Hurst parameter *H* within interval H∈(0,1) to study the time evolution of the number of those infected by the coronavirus in countries where the number of cases is large as Brazil is studied. The rises and falls of novel cases daily or the fluctuations in the official data are treated as a random term in the stochastic differential equation for the fractional Brownian motion. The projection of novel cases in the future is treated as quadratic mean deviation in the official data of novel cases daily since the beginning of the pandemic up to the present. Moreover, the rescaled range analysis (RS) is employed to determine the Hurst index for the time series of novel cases and some statistical tests are performed with the aim to determine the shape of the probability density of novel cases in the future.

## 1. Introduction

The coronavirus disease emerged at the end of 2019 and since then it has become a global crisis. The very quick spreading of the disease made the study of its spreading of great importance for its control and prevention [1,2,3,4,5,6]. On the other hand, the mathematical modeling for the spreading of diseases has been performed since the works of Daniel Bernoulli in the 17th century for spreading of the small pox. Later, the Bernoulli model has been modified with the aim that unbounded growth does not occur and randomness effects are included in the model. At first, a factor was introduced that had the effect of making the variation rate of population growth *N*, negative, being, in this case that the model is given by dN/dt=−αN(1−νN)(1−ηN), where the parameters α and η are in the range α>0 and 0<η<ν. Moreover, taking some randomness in these parameters, we obtain a more realistic model to handle real situations [7,8,9,10,11,12]. In a general way, whether the size of the population at time *t* is N(t), being the relative rate of growth of the population at time *t*, we have the effective population in time *t*, $N_{e}\left(t\right)$, subject to some randomness due to the environmental effects: $N_{e}\left(t\right)=N\left(t\right)+\xi \left(t\right)$, where the first term N(t) is deterministic, while the second term ξ(t) is random (reflecting the environmental randomness effect).

The purpose of this paper is the epidemic modeling of new cases of coronavirus using the nonlinear stochastic differential equation approach with fractional white noise and Hurst parameter at range H∈(0,1), thus showing that the fluctuations of officially released COVID data follow non-trivial self-similar and Gaussian statistics, as a fractional Gaussian driving noise would imply. We analyze the rises and falls of novel cases daily of COVID-19 reported by the public healthy agencies in countries as Brazil, where the low number tests performed in the population lead to a great uncertainty in the official data. Due to the possibility that the dynamics of novel cases might exhibit long-range correlations; thus, it may be described in terms of long-memory processes such as the fractional Brownian motion [13]. Since in many problems related to network traffic analysis, finances and many other fields, the processes under study seem empirically to display self-similar properties and the long-range dependent properties and since the fractional Brownian motions are the simplest processes of this kind, it is important to have a systematic study of these processes and to use them to construct other stochastic processes.

The use of standard stochastic differential equations has already been used in the literature to study the behavior of time series for price dynamics in refs. [14,15,16,17]. In addition, the use of standard Brownian motion that corresponds to the case of Hurt index H=1/2 was employed recently for studying the spreading of COVID-19 in ref. [3]. The stochastic dynamics smoking with non-Gaussian noise was studied in refs. [18,19,20]. The dynamics of a stochastic COVID-19 epidemic model with jump-diffusion was then analyzed in ref. [18]. The stochastic susceptible-infected-recovered (SIR) dynamics expressed using an ordinary Itô-stochastic differential equation has been proposed in refs. [21,22,23,24,25,26,27]. The statistical analysis described by the master equation and transition rates for the infection process was made in ref. [28]. Moreover, the statistical inference in the epidemic model for the Ebola virus was performed in ref. [29]. The discrete-time epidemic model with binomial distribution has been used to study the transmission rate of COVID-19 in [30]. A statistical model for the health care impact of COVID-19 in India has been studied in [31]. The impact of mobility restriction in COVID-19 superspreading was analyzed in [32]. The effect of immunization through vaccination on the susceptible–infected–susceptible (SIS) epidemic spreading model was analyzed in [33]. Here, we propose the analysis based on the fractional Brownian motion to study the dynamics of novel cases. Moreover, we aim to prove that noise has long memory. The plan of this paper is as follows: in Section 2, we describe fractional white noise analysis as a model for dynamics of the spreading of disease. In Section 3, we present the numerical results for the model and the long range memory by rescaled range analysis (RS). In Section 4, we present our conclusions and final remarks.

## 2. Fractional White Noise Analysis

The fractional Brownian motion (BMF) is defined as a family of Gaussian processes, being introduced by Kolmogorov in ref. [34] and indexed by the Hurst parameter *H* into the range H∈(0,1), being the first application made by Hurst in 1951 to model the long-term storage capacity of reservoirs along the Nile River [35,36]. The fractional Brownian motion was defined in refs. [37,38,39], where, for each H∈(0,1), a real-valued Gaussian process $B_{H}\left(t\right)$, t≤0 is defined by $\langle B_{H}\left(t\right)\rangle =0$ and $\langle B_{H}\left(t\right)B_{H}\left(s\right)\rangle =\frac{1}{2}\left[t^{2H}+s^{2H}-{\left(t-s\right)}^{2H}\right]$ for all $s,t\in R_{+}$. If H=1/2, the fractional Brownian motion is the standard Brownian motion or the Wiener process. We consider *H* restricted to range H∈(0,1) and apply this equation as well as its modifier for the infected numbers by coronavirus N(t) in each day. Hence, we use the nonlinear fractional stochastic equation with a drift term in the form of a logistic model with a random term to perform the analysis of the spreading of novel cases of coronavirus N(t), given by

(1) $$\begin{array}{c} dN\left(t\right)=f\left(t\right)dt-\alpha \left(t\right)N\left(t\right)\left(1-\nu (t)N(t)\right)\left(1-\eta (t)N(t)\right)dt\\ +\beta \left(t\right)⋄dB_{H}\left(t\right), \end{array}$$

where the drift term, f(t), is a better adjustment using a third order polynomial as f(t) = −30,827 $+1177t-8.0186t^{2}+0.02t^{3}$ in the later considered cases obtained by least squares fit to the data. The add of the least square fit f(t) to the logistic model with noise is to take into account the isolation measures and slow vaccination of the populations of each country, which smooths the curve of the growing of novel cases. Due to this, the data are better fitted with a polynomial than fitting directly the solution of the model without noise.

Whether $t\to B_{H}\left(t\right)$ is differentiable into the Schwartz space S(R) of rapidly decreasing smooth functions, ${\left(S_{H}\right)}_{H}^{*}$ being the dual of ${\left(S\right)}_{H}$

(2) $$\frac{dB_{H}\left(t\right)}{dt}=W_{H}\left(t\right),\;\mathrm{in}\;{\left(S\right)}_{H}^{*},$$

where $W{\left(t\right)}_{H}$ is a fractional white noise defined by

(3) $$W_{H}=\sum_{i=1}^{\infty }\left[\int_{R}e_{i}\left(v\right)\phi \left(t,v\right)dv\right]H_{\varepsilon (i)}\left(\omega \right)$$

where ${\left\{e_{n}\right\}}_{n=1}^{\infty }$ is the orthonormal basis of $L_{\phi }^{2}\left(R\right)$ and

(4) $$H_{\varepsilon (i)}\left(\omega \right)=\int_{R}e_{i}\left(t\right)dB_{H}\left(t\right).$$

The $B_{H}$-integration theory of Equation (1) is based on using ordinary products path-wise and leads to an integral that, for integrands g(t,ω), is defined by

(5) $$\begin{array}{c} \int_{a}^{b}g\left(t,\omega \right)\delta B_{H}\left(t\right)=\lim_{|\Delta |\to 0}\sum_{k=0}^{n-1}g\left(t_{k},\omega \right)\left[B_{H}\left(t_{k+1}\right)-B_{H}\left(t_{k}\right)\right], \end{array}$$

where Δ: $a=t_{0}<t_{1}<\cdot \cdot \cdot <t_{n}=b$ is a partition of the interval [a,b] and $\left|\Delta \right|=\max_{0\leq k\leq n-1}\left(t_{k+1}-t_{k}\right)$. These integrals do not have an expectation zero and are called fractional path-wise integrals. Here, we consider the $B_{H}$-integral considered in ref. [40], defined by

**Definition** **1.** Suppose $g:R\to {\left(S\right)}_{H}^{*}$ is a given function such that $g\left(t\right)⋄W_{H}\left(t\right)$ is integrable in ${\left(S\right)}_{H}^{*}$. Then, we define the fractional stochastic integral of Itô type, $\int_{R}g\left(t\right)dB_{H}\left(t\right)$, by

(6) $$\begin{array}{c} \int_{R}g\left(t\right)dB_{H}\left(t\right):=\int_{R}g\left(t\right)⋄dW_{H}\left(t\right). \end{array}$$

*For instance, suppose*

(7) $$\begin{array}{c} g\left(t\right)=\sum_{k=1}^{n}g_{k}\left(\omega \right)\chi_{\left[t_{k},t_{k+1}\right)}\left(t\right),\;g_{k}\in {\left(S\right)}_{H}^{*}, \end{array}$$

*where*

(8) $$\begin{array}{c} \chi_{\left[0,t\right]}\left(\tau \right)=\{\begin{array}{c} 1\;\mathrm{if}\;0\leq \tau \leq 1,\\ -1\;\mathrm{if}\;t\leq \tau \leq 0,\;\mathrm{except}\;t=\tau =0\\ 0\;\mathrm{otherwise}. \end{array} \end{array}$$

**Figure 1:**
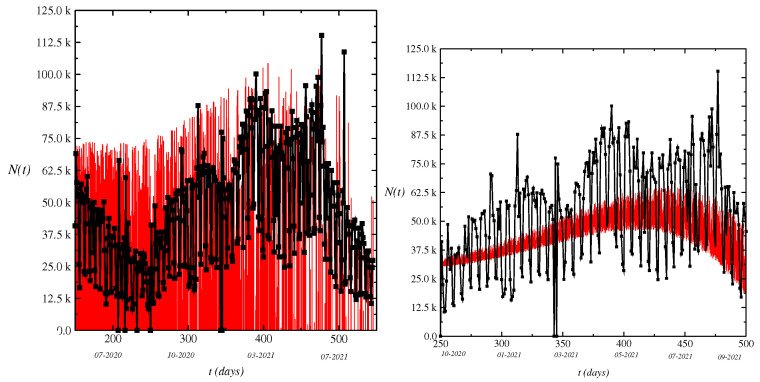
Dynamics of novel cases N(t) in Brazil. The zigzag behavior of the results is reflected by the stochastic term in Equation (1). We plot the time series of the model Equation (1) for a value of Hurst parameter H>1/2 such as H=0.55 (above) and H<1/2 as H=0.30 (under). The black squares are the daily novel cases reported by the Ministry of Health and the red-line is the adjusting of the model Equation (1).

We concentrate on the $B_{H}$-integral given in [39]

(9) $$\begin{array}{c} \int_{R}g\left(t\right)dB_{H}\left(t\right)=\sum_{k=0}^{n}g_{k}\left(\omega \right)⋄\left[B_{H}\left(t_{k+1}\right)-B_{H}\left(t_{k}\right)\right], \end{array}$$

where the definition here is an extension of the fractional Itô integral introduced in ref. [37]. Hence, we concentrate on the $B_{H}$-integral defined by

(10) $$\begin{array}{c} \int_{a}^{b}g\left(t,\omega \right)dB_{H}\left(t\right)=\lim_{|\Delta |\to 0}\sum_{k=0}^{n-1}g\left(t_{k},\omega \right)⋄\left[B_{H}\left(t_{k+1}\right)-B_{H}\left(t_{k}\right)\right], \end{array}$$

where ⋄ denotes the Wick’s product [37,38,39,40,41,42,43,44]. These integrals use the Wick’s products and are known as the fractional Itô integrals with reference to the corresponding situation for standard Brownian motion [38,40]. We consider the white noise calculus based on $B_{H}\left(t\right)$, H∈(0,1). Due to the behavior of novel cases, we consider the deterministic part adjusting to the daily novel cases using a suave curve. The aim is simply to provide a smoothed version of the time series using standard methods as the lowest regression. Furthermore, we aim to calculate the ensemble of all possible trajectories using the fractional Brownian motion as is made in Figure 1.

The zigzag behavior in the range of large *t* values reflects in an increase of the uncertainty in the data and low-number of test performed in the population. For modeling of the zigzag behavior of novel cases, we add a random noise term in the logistic model for the spreading of an infectious number as given by Equation (1) in order to simulate the effect of rises and falls of novel cases [3]. The α, ν, and η parameters comes from the logistic model with a threshold. We use the values obtained by Bernoulli for smallpox α=ν=0.13; however, we should have α(t) and ν(t). The range of η(t) values is into 0<η(t)<ν(t). f(t) comes from the adjustment of least squares to the data into the period considered. The daily fluctuations with a weekly cycle of reported cases have been observed in many countries and are due to the diagnostic and data reporting practices [45]. We obtain the time series of the model Equation (1) using the values obtained by Bernoulli given by α=ν=0.13, η=0.063. Furthermore, we use a low intensity of fractional white noise β as $\beta =1.0\times 10^{-6}$.

## 3. Numerical Results

We write the BMF model that is a Gaussian process $W_{H}\left(t\right)$, t>0 with zero mean and stationary increments and variance given by $\langle W_{H}^{2}\left(t\right)\rangle =t^{2H}$. The sample path of the BMF model is a fractal curve with fractal dimension d=1/H, where H=1/2 corresponds to the standard Brownian motion whose increments $\Delta W_{H}\left(t\right)=W_{H}\left(t+1\right)-W_{H}\left(t\right)$ are statistically independent. For H≠1/2, the increments $\Delta W_{H}\left(t\right)$ are known as fractional white noise, showing long-range correlation as $\langle \Delta W_{H}\left(t+h\right)\Delta W_{H}\left(t\right)\rangle ≃2H\left(2H-1\right)h^{2H-2}$, for h→∞. For H∈(1/2,1), the increments are positively correlated, thus exhibiting a persistent behavior. For H∈(0,1/2), the increments are negatively correlated, showing anti-persistence. For H=1/2, we write the Wiener increment as $dW_{H}\left(t\right)∼{\left(\sqrt{dt}R_{G}\right)}^{2H}$, where $R_{G}$ is an aleatory generator number with Gaussian distribution of mean zero and variance $\sigma_{W_{H}}^{2}=1$. In the following, we write Equation (1) in the discrete form with the time step Δt used in the simulations as Δt=0.001, with *t* running along chosen units (say seconds, minutes, or days). In Figure 1, we plot the time series of the model Equation (1) for values of noise intensity as $\beta \left(t\right)=1.0\times 10^{-6}t^{3}$. We have the time series of the variation of novel cases oscillating quickly as displayed in the figure. The results of simulations show that value of *H* of 0.3 (H≤0.5) is insufficient for good accuracy of the model and the value of H=0.55 looks better and H=0.46789 may be quite suitable for a best fit if the value of *H* does not depend on the time, which is probably not the case. We plot the time series of the model for values of Hurst parameter H>1/2: H=0.55. The results for H=1/2 correspond to random walk [3]. The intensity of the value of fractional white noise used in the calculations is such as $\beta \left(t\right)=1.0\times 10^{-6}t^{3}$.

**Figure 2:**
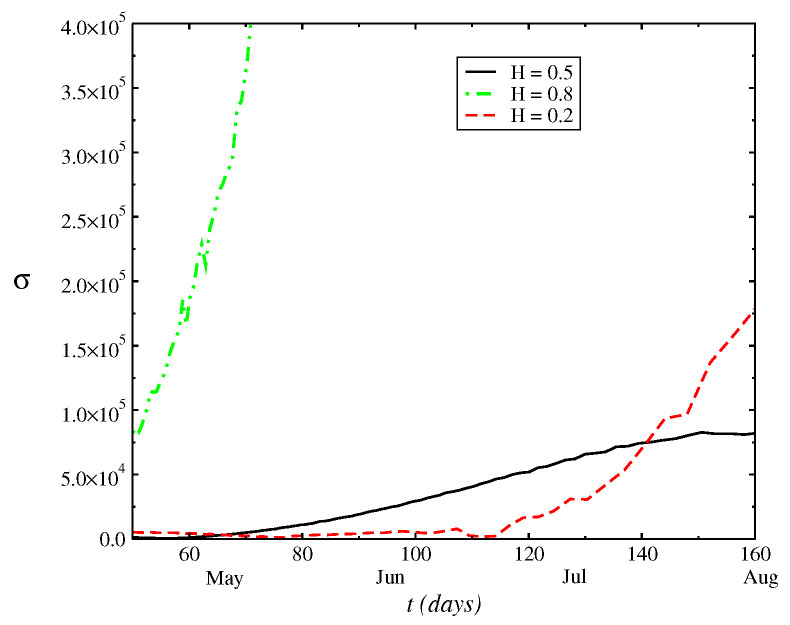
Behavior of half-width of the distribution as a function of *t*, σ(t). The half-width gives an expectation of novel cases in each day *t*.

**Figure 3:**
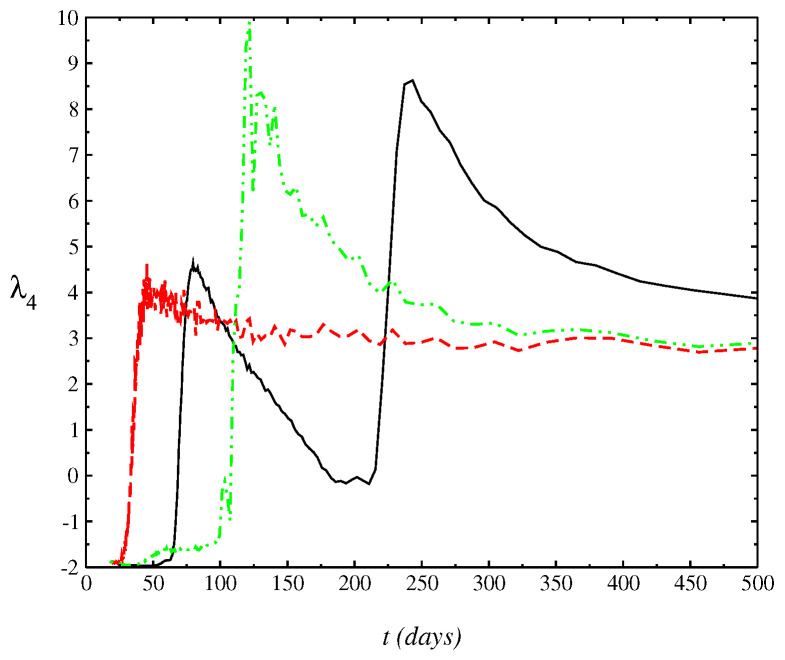
Behavior of the kurtosis as a function of *t*, $\lambda_{4}\left(t\right)$ for different values of Hurst index: H=0.5 (black-solid line), H=0.2 (dashed-red line), and H=0.8 (dot-dashed-green line), that is, for a value above and below the value H=0.5, which corresponds to the standard Brownian motion. The range of negative values gives an estimating of the shape of distribution which becomes closest to a Gaussian for $\lambda_{4}=0$ at a range of large *t* values since the first cases reported.

In Figure 2, we plot the numerical result of the half-width of the distribution of N(t) as a function of time *t*, σ(t). We calculate the variance of the distribution where the standard deviation gives an estimation of novel cases in each day *t*. The range of data considered here is from 14 March 2021 up to 21 July 2021. The difference to the real data is due to the approach used. We calculate the *n*th order moments $\mu_{n}=\langle {\left(x-m_{1}\right)}^{n}\rangle$ until the fourth order about the mean or central moments, where we have the following relations: $c_{1}=\mu_{1}$, $c_{2}=\mu_{2}$, $c_{3}=\mu_{3}$, $c_{4}=\mu_{4}-3\mu_{2}^{2}$. Normalized measures commonly used indicating a deviation from the Gaussian are the kurtosis $\lambda_{4}$, defined as $\lambda_{4}=\left(\mu_{4}/\sigma^{4}\right)-3$ and the skewness, $\lambda_{3}$. In Figure 3, we display that the behavior of $\lambda_{4}\left(t\right)$. $\lambda_{4}\left(t\right)$ is numerically calculated by solving Equation (1)). Moreover, the kurtosis relates to the deviation of the tail of the distribution as compared to Gaussian $P\left(N\left(t\right),t\right)=\frac{1}{\sqrt{4\pi \beta (t)}}e^{-{\left[N\left(t\right)\right]}^{2}/4\beta \left(t\right)}$, whose solution would correspond to Equation (1) for ν(t)=η(t)=0 and f(t)≡0. The range of negative values obtained for the kurtosis indicates that the shape of the distribution is closest to Wigner’s distribution [11]. Furthermore, at range of small *t* considered, where the kurtosis is close to zero, we have that the distribution is closest to a Gaussian ($\lambda_{4}=0$).

### Rescaled Range Analysis

**Figure 4:**
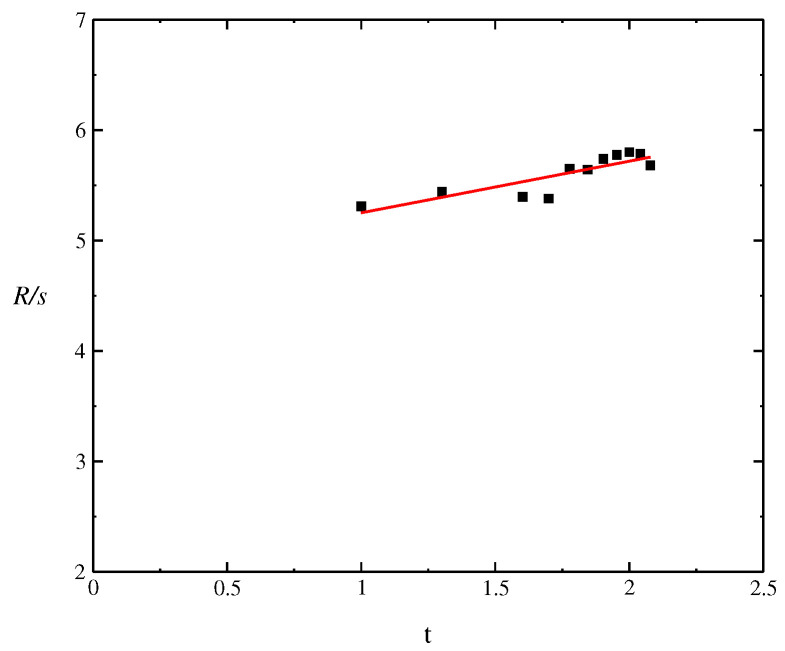
Log-Log graphic to determine the Hurst index using the rescaled range (RS) method.

There are several estimators for the Hurst exponent, such as rescaled range (RS) analysis and multifractal and detrended fluctuation analysis [46,47] (DMFA) and (DFA) that differ basically by the choice of the fluctuation measure. The obtained exponent by DMFA and DFA is similar to the Hurst exponent, except that both DMFA and DFA may also be applied to signals who have underlying statistics. In Figure 4, we present the Log-Log graphic with the objective of determining the Hurst exponent. The window of data considered for analysis were the last 120 days before 9 January 2021. We use the RS analysis, where the Hurst exponent is used as a measure of long-term memory of time series. It relates to the autocorrelations of the time series and the rate at which these decrease as the lag between pairs of values increases. The RS analysis is the oldest and best-known method to estimate the Hurst indexes. In general, the RS analysis is an estimator of the Hurst exponent for random walks; however, in this nonlinear logistic dynamic, the rescaled range is a reliable exponent. The start point of the method is to determine the standard deviation using the mean field approach as $\sigma =\sqrt{\langle N^{2}\rangle -{\langle N\rangle }^{2}}$ and the range *R* as

(11) $$\begin{array}{c} R={\left[\sum_{t=1}^{n}N_{t}-n\langle N\rangle \right]}_{max}-{\left[\sum_{t=1}^{n}N_{t}-n\langle N\rangle \right]}_{min}\\ t=1,2,...,n. \end{array}$$

The rescaled range series (RS) is given by

(12) $$\begin{array}{c} \left(R/s\right)=\frac{R_{t}}{\sigma },\;\log\left(\frac{R_{t}}{\sigma }\right)=H\log\left(t\right), \end{array}$$

where *H* is the Hurst exponent. We obtain the value of the Hurst exponent using the RS method as H=0.4678(9). The presented numerical results show the approximation of the Hurst parameter *H* close to H=1/2, which is related to the classical Brownian motion and common distribution shape parameter as moments, kurtosis, and skewness. Although the value obtained for the data set is very close to 0.5, we should have *H* time dependent, H(t), that is, the estimator is relevant in the current context and may be time dependent. The value found for the data set is within the interval H∈(0,0.5), thus indicating an anti-persistence for the time series of case numbers N(t) reported daily.

## 4. Conclusions

In brief, we analyzed the spreading of novel cases of coronavirus using the nonlinear stochastic differential equation with fractional white noise (nonlinear fractional Brownian motion approach) to model the rises and falls of novel cases of coronavirus in countries such as Brazil. The variability in the time series, for instance in Brazil, is mostly due to the case reports not being filed on weekends, leading to a large-amplitude oscillation within a period of seven days. Furthermore, the uncertainty in the results generated by the low number of tests performed in the populations and to underreporting generates a large uncertainly in the official results and, consequently, the addition of randomness in the differential equations becomes necessary for the growth of infected numbers. We find a small deviation in the Hurst indexes from H=1/2 as H≈0.47, indicating a small deviation from the standard Brownian motion approach. An investigation into whether fractional Gaussian noise is indeed the best (phenomenological) explanation for the fluctuations of COVID data can be made in a future work. The starting point would be more established schemes such as DFA, with as few free parameters as possible, and a much larger database, i.e., COVID data from many countries. However, we must be careful in using fractional, i.e., Gaussian noise with algebraic correlation, and further investigate in a data-driven manner what correlation structure the fluctuations display across different data sets.
